# Fe‐Substituted MoO*_x_* Catalysts With Lattice Distortion–Vacancy Coupling for Enhanced Alkaline Oxygen Evolution Reaction

**DOI:** 10.1002/cssc.202502390

**Published:** 2026-02-12

**Authors:** Minhui Kim, Byounguk Yu, Hye Young Koo, Yuchan Kim, Dahee Park

**Affiliations:** ^1^ Energy and Environment Materials Research Division Korea Institute of Materials Science (KIMS) Changwon Republic of Korea; ^2^ Nano Materials Research Division Korea Institute of Materials Science (KIMS) Changwon Republic of Korea

**Keywords:** catalyst modification, lattice oxygen mechanism, molybdenum oxide, oxygen evolution reaction

## Abstract

Molybdenum oxide catalysts have been widely investigated as cost‐effective electrocatalysts for water electrocatalysis due to their easily tunable electronic structures. However, their oxygen evolution reaction (OER) activities remain limited by their low electrical conductivities and electronically inactive oxidation states, thereby prompting the development of various alternative strategies. Herein, Fe‐substituted MoO_*x*_ catalysts with controlled lattice distortion and oxygen vacancy concentrations are proposed. Fe‐substituted MoO_*x*_ is synthesized via aerosol spray pyrolysis and subsequent postannealing to control its interfacial properties. Fe substitution induces spatial segregation from Mo, leading to the formation of a yolk–shell structure that exposes abundant active sites. Furthermore, Mo orbital hybridization improves the electronic structure and greatly enhances electrical conductivity. The optimized yolk–shell‐structured FeMoO_*x*_ catalyst exhibits excellent performance at a high current density of 100 mA cm^−2^, delivering a low overpotential of 294 mV and maintaining stable performance over 100 h. In situ electrochemical analyses reveal that temperature control of the charge distribution enhances oxygen intermediate adsorption and promotes O—O bond formation through lattice oxygen species, thereby activating the lattice oxygen mechanism. This study provides mechanistic insights and a practical design strategy toward developing cost‐effective, high‐performance OER electrocatalysts based on transition‐metal‐modified molybdenum oxides.

## Graphical abstract

Fe–MoOx electrocatalysts with controlled lattice distortion and oxygen vacancies were synthesized via one‐step spray pyrolysis. Temperature‐regulated Fe–Mo charge redistribution promoted orbital hybridization and activated the lattice oxygen mechanism. The yolk–shell structure achieved high activity and durability under high‐current operation, addressing the key limitation that has hindered MoOx‐based catalysts in oxygen evolution reactions.

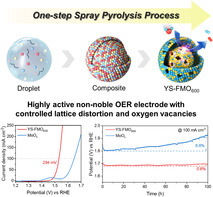

## 1 Introduction

Hydrogen production via water electrolysis has attracted considerable attention as a sustainable and carbon‐free protocol for generating clean hydrogen [1, 2, 3]. In particular, electrocatalytic water splitting under alkaline conditions offers advantages such as high‐current‐density operation and the use of cost‐effective materials [4, 5, 6]. However, the sluggish kinetics and high overpotential of the oxygen evolution reaction (OER) at the anode remain major bottlenecks, severely limiting the overall efficiency of the electrolysis process. It is therefore essential to develop high‐performance OER electrocatalysts with both high activity and long‐term stability.

Specifically, molybdenum‐based materials have attracted attention as potential electrocatalysts for water electrolysis, owing to their low cost and tunable electronic structures derived from their spatially extended 4d orbitals [7, 8]. Recently, molybdenum oxides, such as MoO_2_ and MoO_3_, have been extensively investigated as OER catalysts owing to their high electrical conductivities and strong catalytic reactivities at the edge sites [9, 10, 11, 12]. However, the low electrical conductivity and inability of fully oxidized Mo^6+^ species to provide active sites for oxygen intermediate adsorption significantly limit their application as OER catalysts based on single‐metal oxides. To overcome these intrinsic limitations, researchers have employed a range of strategies (e.g., metal substitution, single‐atom doping, and heterojunction engineering), thereby enhancing the electrochemical performance of molybdenum oxides [9, 13, 14, 15].

Recently, it has been demonstrated that MoO_*x*_ exhibits strong binding with oxygen intermediates (*O), thereby restricting its application as an OER electrocatalyst [10, 16]. The incorporation of secondary transition metals has therefore been proposed as an effective strategy to optimize the adsorption energies of the oxygen intermediates [17, 18, 19, 20, 21]. Such modification regulates the oxygen vacancy (O_v_) content and induces electron redistribution, shifting the O 2p level closer to the Fermi level [22, 23]. Additionally, an enhanced degree of M−O hybridization induces the partial occupation of antibonding states, ultimately weakening the M—O bond. This promotes lattice oxygen redox reactions and activates the lattice oxygen mechanism (LOM) [24, 25].

Many researchers have also attempted to use electron‐rich 3d transition metals (including Fe, Ni, and Co) as effective dopants to boost OER activity. This is achieved by modulation of the adsorption energies and bonding strengths of the oxygen intermediates, while simultaneously improving the electrical conductivity of the catalyst [26, 27, 28]. Moreover, orbital hybridization between Mo and different transition metals has been demonstrated to enhance the accessibility of the oxygen nonbonded states, which may facilitate the LOM pathway during the OER [29, 30].

In this study, an Fe‐substituted MoO_*x*_ catalyst is developed that remains stable under severe high‐current conditions by exploiting the synergistic effects of O_v_ and lattice distortion, coupled with structural control through variation in the annealing temperature. Fe‐substituted MoO_*x*_ is synthesized via a one‐step spray pyrolysis process by partially substituting Fe for Mo species. Additionally, the influence of the annealing temperature on the surface segregation behavior and metal–metal synergistic effects is investigated to elucidate the OER mechanism. It is proposed that the simultaneous tuning of the lattice distortion and the O_v_ concentration will provide a simple yet effective pathway for developing high‐performance Mo‐based OER catalysts, while offering insights into the rational design of transition metal‐substituted molybdenum oxides.

## 2 Results and Discussion

**SCHEME 1:**
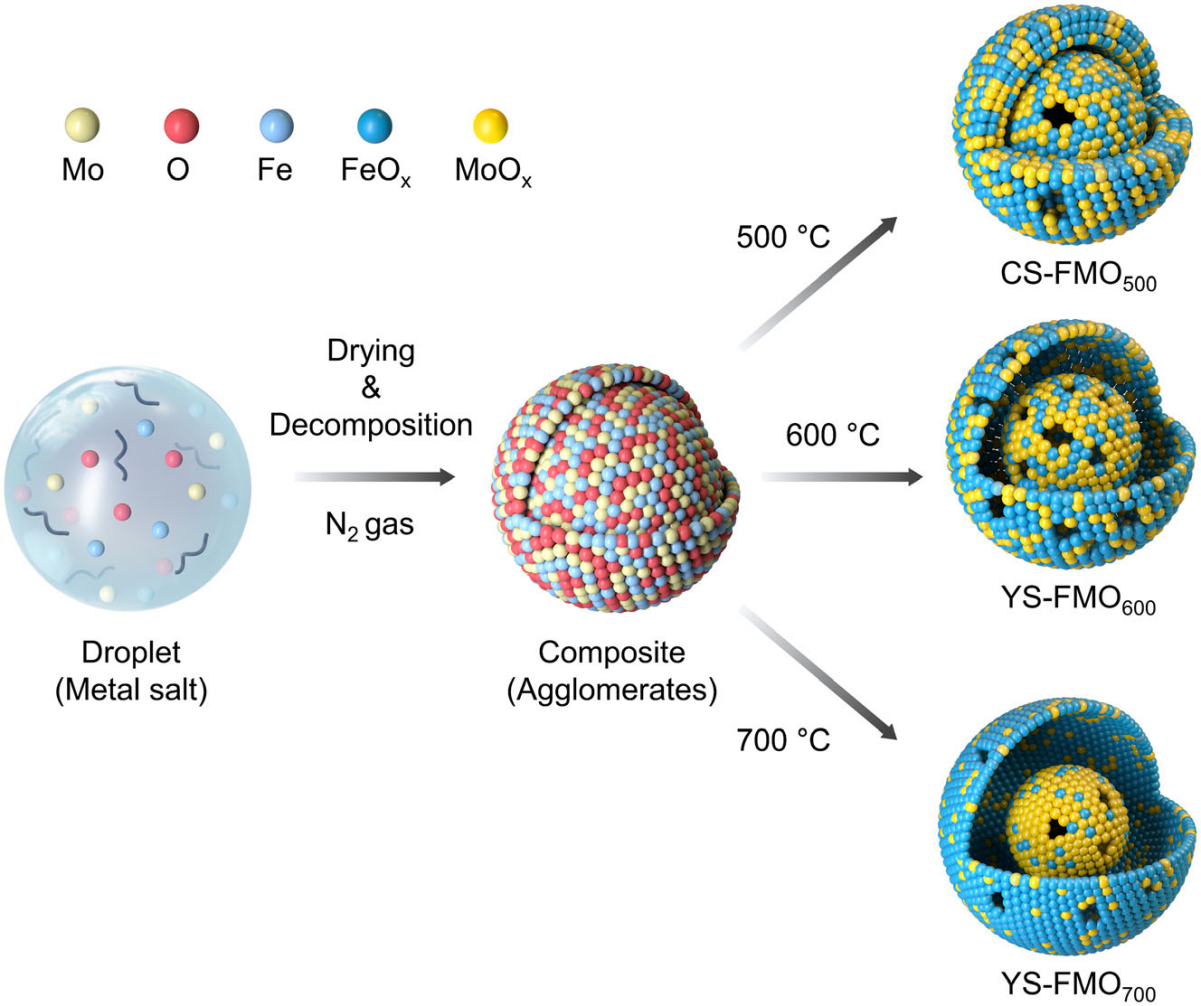
Schematic representation of the synthetic process employed to obtain the CS‐FMO_500_, YS‐FMO_600_, and YS‐FMO_700_ systems.

The Fe‐substituted MoO_*x*_ catalysts were synthesized via one‐step spray pyrolysis, as outlined in Scheme 1. Specifically, the precursor solution was sprayed and evaporated into fine aerosols then thermally decomposed by pyrolysis to form particles [31, 32, 33]. The resulting Fe‐substituted MoO_*x*_ catalysts were annealed at different temperatures (500°C, 600°C, and 700°C) to control their morphological and electrical properties. Consequently, the CS‐FMO_500_ catalyst (annealed at 500°C) exhibited a core–shell Fe‐substituted MoO_*x*_ structure, while the YS‐FMO_600_ and YS‐FMO_700_ catalysts (annealed at 600°C and 700°C, respectively) displayed yolk–shell Fe‐substituted MoO_*x*_ architectures. During the annealing process, partial removal of lattice oxygen from MoO_3_ is expected to generate O_v_, which can serve as effective active sites for electrocatalysis [10]. The incorporation of transition metal species is anticipated to induce structural defects in the MoO_3_ lattice, further generating structural and electronic correlations that enhance the electrocatalytic activity [34, 35, 36].

**FIGURE 1:**
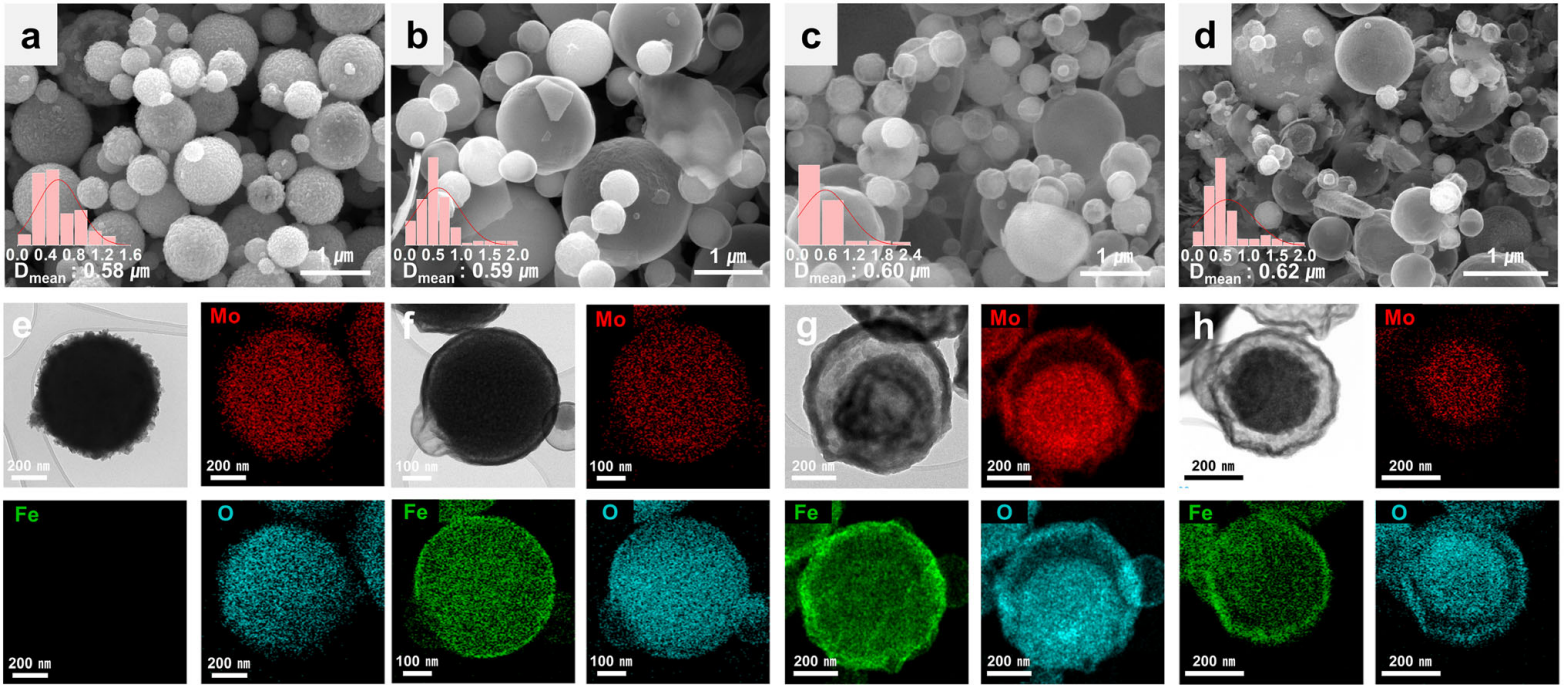
Morphological properties of the Mo‐based catalysts. (a–d) FE‐SEM images of (a) MoO_*x*_, (b) CS‐FMO_500_, (c) YS‐FMO_600_, and (d) YS‐FMO_700_. The insets show the particle size distribution. (e–h) TEM images and corresponding STEM‐EDS elemental mapping images of (e) MoO_*x*_, (f) CS‐FMO_500_, (g) YS‐FMO_600_, and (h) YS‐FMO_700_, showing Mo (red), Fe (green), and O (cyan).

The synthesized catalysts exhibited distinct optical and structural characteristics based on the degree of Fe substitution and the annealing temperature (Figure S1). The field‐emission scanning electron microscopy (FE‐SEM) image of MoO_*x*_ in Figure 1a reveals spherical particles with rough surfaces and an average diameter of ∼580 nm. The FMO samples exhibited relatively smooth yet porous surfaces, and their apparent particle size increased in the order YS‐FMO_700_ > YS‐FMO_600_ > CS‐FMO_500_ (Figure 1b–d). The fine morphologies and elemental compositions were characterized by high‐angle annular dark‐field scanning transmission electron microscopy (HAADF–STEM) and energy‐dispersive X‐ray spectroscopy (EDS). The MoO_*x*_ sample exhibited a dense solid structure with uniformly distributed Mo species (Figure 1e). CS‐FMO_500_ exhibited a core–shell structure with Fe migrating toward the periphery (Figures 1f and S2). During the annealing process, the difference in surface energies between the Fe‐ and Mo‐based species drives atomic rearrangement to minimize the interfacial free energy [37, 38]. Consequently, Fe shows a tendency to segregate toward the surface, and this behavior becomes more pronounced at higher annealing temperatures. A yolk–shell structure was observed for the YS‐FMO_600_ catalyst (Figure 1g) with the remaining Fe ions being found in the core region. The YS‐FMO_700_ catalyst featured a more pronounced structural contrast between the core and the shell, with the majority of Fe ions migrating to the shell (Figure 1h). The porous yolk–shell architecture enhances electrolyte accessibility and increases the electrochemically active surface area [39, 40]. In addition, an appropriate degree of Fe surface segregation promotes the formation of Fe–O–Mo hetero‐sites at the shell region, which is expected to contribute to the improved OER performance [41].

Brunauer–Emmett–Teller (BET) analysis was employed to investigate the specific surface areas of the yolk–shell structure for YS‐FMO_600_ and the bare MoO_*x*_, which were synthesized at the same annealing temperature (Figure S3). YS‐FMO_600_ presented a specific surface area of 17 m^2^ g^−1^ and a mesopore distribution of 24.56 nm, which is significantly higher than the corresponding values for MoO_*x*_, namely a specific surface area of 9.9 m^2^ g^−1^ and a micropore size of 0.73 nm. This confirms that Fe incorporation creates a larger active surface area and enables efficient contact with the active species in the electrolyte [42, 43].

**FIGURE 2:**
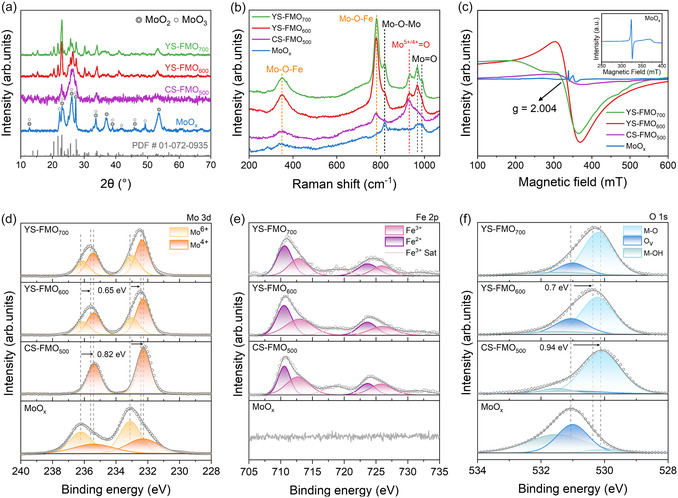
Electronic structure analysis of the catalysts. (a) XRD patterns, (b) Raman spectra of MoO_*x*_, and (c) EPR spectra recorded for CS‐FMO_500_, YS‐FMO_600_, and YS‐FMO_700_. (d–f) XPS spectra for the (d) Mo 3d, (e) Fe 2p, and (f) O 1s components of the Mo‐based catalysts.

The crystal structures of the MoO_*x*_ and FMO series (i.e., CS‐FMO_500_, YS‐FMO_600_, and YS‐FMO_700_) catalysts were subsequently characterized using X‐ray diffractometry (XRD). For MoO_*x*_, the diffraction peaks were indexed to MoO_2_ (PDF# 01‐076‐1807) and MoO_3_ (PDF# 00‐047‐1320), indicating that MoO_*x*_ possesses a mixed polycrystalline structure (Figure S4). After Fe substitution, characteristic diffraction peaks corresponding to Fe_2_(MoO_4_)_3_ (PDF# 01‐072‐0935) were observed, indicating transition to a single‐phase structure (Figure 2a). The incomplete crystallization of CS‐FMO_500_ was attributed to the limited phase transition at this low annealing temperature, resulting in low diffraction intensities and broadened peaks [44, 45]. To further elucidate the local crystal structure, high‐resolution transmission electron microscopy (HRTEM) analysis was performed. For MoO_*x*_, lattice fringes corresponding to the (011) and (040) planes of MoO_2_ and the (110) plane of MoO_3_ are clearly observed (Figure S5a). The FMO series exhibits lattice fringes associated with the Fe_2_(MoO_4_)_3_ crystal structure, while variations in d‐spacing among the samples reflect lattice distortion and localized strain induced by differences in the synthesis temperature (Figure S5b–d) [46, 47].

Raman spectroscopy was subsequently employed to investigate the changes in the bonding environment [36, 48, 49]. As shown in Figure 2b, the emergence of peaks at ∼350 and ∼784 cm^−1^ suggests the formation of Fe–O–Mo linkages, indicating that Fe incorporation directly modifies the MoO_4_ coordination environment. The peak associated with the asymmetric Mo–O–Mo stretching vibration at ∼821 cm^−1^ decreases in intensity upon Fe substitution, but reappears in the spectrum of YS‐FMO_700_ as the crystallinity of Fe_2_(MoO_4_)_3_ increases. The peaks observed in the 930–990 cm^−1^ region are assigned to the symmetric M=O stretching band. Notably, the band in the 960–990 cm^−1^ region gradually split into signals at 968 and 991 cm^−1^ upon increasing the annealing temperature, indicating that higher temperatures further intensified the extent of bond asymmetry and lattice distortion [50]. The structural defects were further examined by electron paramagnetic resonance (EPR) spectroscopy, and the results are presented in Figure 2c. MoO_*x*_ exhibited a sharp and intense signal at *g* ≈ 2.004, attributed to the localization of unpaired electrons at the O_v_. Additionally, the reduction of MoO_3_ resulted in the appearance of a broad signal attributed to Mo^5+^ species at 370 mT (*g* ≈ 1.94) [51, 52]. YS‐FMO_600_ displays a broader EPR line shape than MoO_*x*_, reflecting its enhanced paramagnetism and spin inhomogeneity [53]. For YS‐FMO_700_, the EPR spectrum shows a more pronounced distortion, likely due to enhanced lattice asymmetry and spin disorder [20, 54]. These spectral features are consistent with the structural distortion observed in the Raman analysis.

The chemical states and electronic interactions of the catalysts were analyzed using X‐ray photoelectron spectroscopy (XPS), with all catalysts exhibiting Mo 3d peaks corresponding to Mo^6+^ and Mo^4+^ species at 233.1 and 232.3 eV, respectively [55, 56]. In the case of MoO_*x*_, the Mo 3d spectrum revealed Mo^6+^ and Mo^4+^ atomic ratios of 33.8% and 23.4%, respectively, indicating that Mo is present in its highest oxidation state in this sample (Figure 2d and Table S1). However, the incorporation of Fe with a relatively lower electronegativity induces electron transfer to Mo, leading to a pronounced change in the Mo chemical state [57, 58]. For CS‐FMO_500_, a negative shift of 0.82 eV was observed for the Mo 3d peak, and the Mo^4+^ fraction increased to 59.1%, indicating substantial reduction of the Mo species. Upon increasing the annealing temperature, the Mo^4+^ content gradually decreased to 39.8% and 37.7% for YS‐FMO_600_ and YS‐FMO_700_, respectively. Additionally, the Fe 2p spectrum displayed two characteristic peaks at 712.7 and 710.6 eV, corresponding to Fe^3+^ and Fe^2+^, respectively [59, 60]. Although all three catalysts possessed similar Fe^2+^/Fe^3+^ ratios, slight changes in the oxidation states were observed due to electron transfer to Mo. For example, CS‐FMO_500_ was found to possess a higher Fe^3+^ content than the other samples, as detailed in Figure 2e and Table S2. These findings indicate that electron redistribution among transition metals is highly sensitive to temperature, consistent with the thermally induced spin−state transition of Fe, which ultimately modulates the oxidation states and charge transfer at elevated temperatures [61]. Furthermore, the O 1s spectra contain signals corresponding to M—OH, M—O_v_, and M—O bonds [62]. In the case of MoO_*x*_, the reduction of Mo^6+^ to Mo^4+^ promoted O_v_ formation due to oxygen loss from the Mo—O—Mo lattice (Figure 2f and Table S3) [63]. The obtained results clearly indicate that Fe substitution leads to a decrease in the number of Mo–O–Mo bonds, while O_v_ formation was significantly regulated by the annealing temperature [10, 64]. As presented in Figure S6, the O_v_ content of the FMO series increases in the order CS‐FMO_500_ < YS‐FMO_700_ < YS‐FMO_600_. The optimized electronic interactions and O_v_ contents are therefore expected to modulate the adsorption energies of the OER intermediates and suppress structural disintegration of the catalyst, thereby enhancing its activity and durability [65, 66].

As previously reported, Fe substitution effectively modulates the electronic structure of Mo and influences the electrode bandgap [67]. Thus, ultraviolet–visible (UV–vis) spectrophotometry was conducted over a wavelength range of 200–700 nm to estimate the bandgap energies of YS‐FMO_600_ and MoO_*x*_ (Figure S7). As shown in Figure S5b, the bandgap of YS‐FMO_600_ (1.84 eV) is markedly lower than that of MoO_*x*_ (2.19 eV), demonstrating that Fe substitution greatly improves the conduction properties of MoO_*x*_ [30, 68].

**FIGURE 3:**
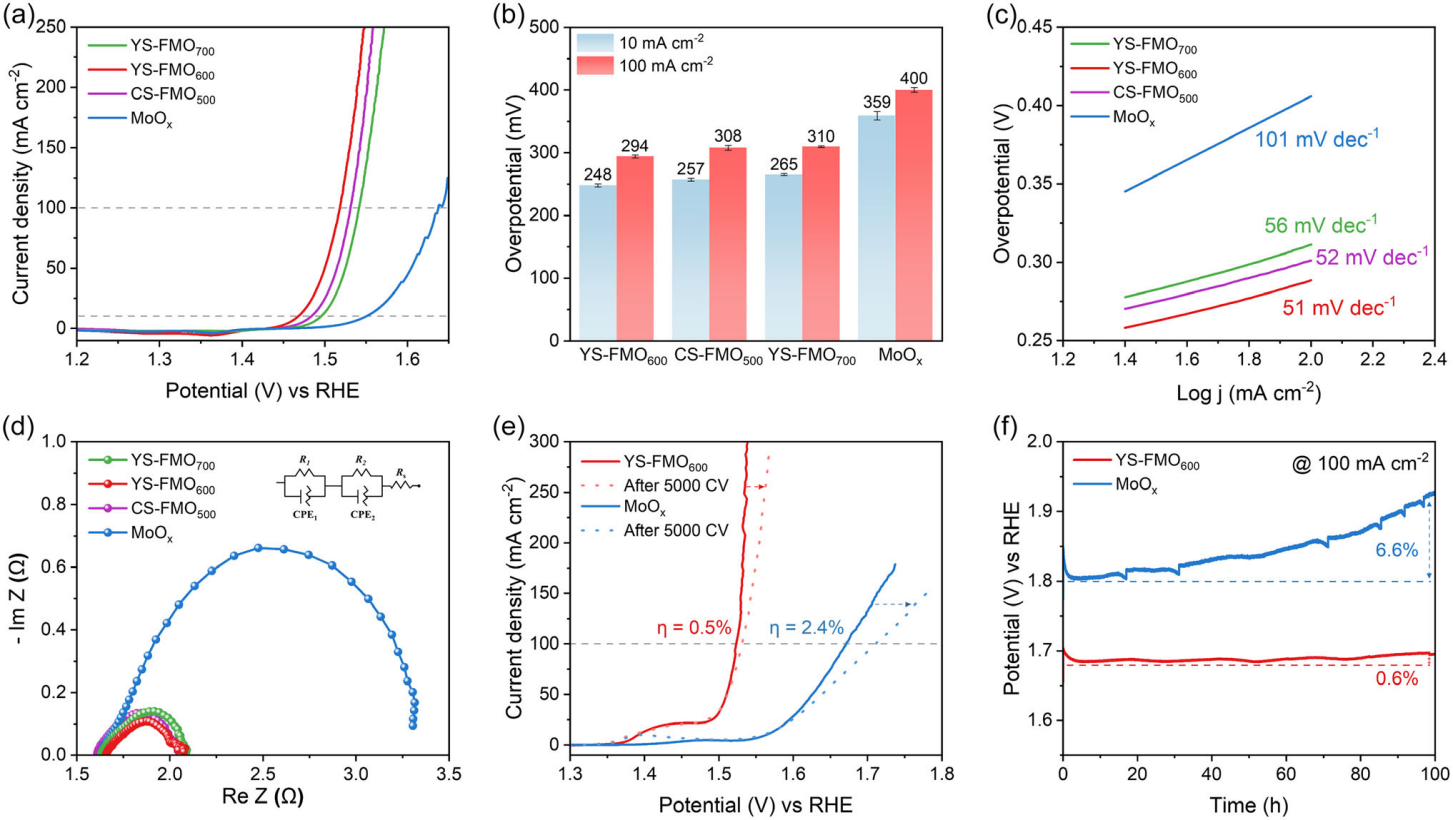
Electrocatalytic OER performances of the catalysts in a 1 M KOH electrolyte. (a) LSV curves with iR correction. (b) Overpotential values recorded at 10 and 100 mA cm^−2^. (c) Tafel slopes and (d) EIS profiles recorded at 1.6 V_RHE_ for MoO_*x*_, CS‐FMO_500_, YS‐FMO_600_, and YS‐FMO_700_. (e) LSV curves recorded after 5000 cycles of the accelerated durability test. (f) CP results obtained at 100 mA cm^−2^ over 100 h (without iR compensation) for YS‐FMO_600_ and MoO_*x*_.

The electrocatalytic properties of the FMO series and MoO_*x*_ samples were systematically investigated by linear sweep voltammetry (LSV) in a conventional three‐electrode system with a 1 M KOH electrolyte. Additionally, iR correction was applied based on the solution resistance obtained from electrochemical impedance spectroscopy (EIS) measurements performed at the open‐circuit potential [69]. As presented in Figure 3, the LSV and EIS measurements provide key activity parameters, including the overpotentials at 10 and 100 mA cm^−2^, Tafel slopes, and stability after repeated cyclic voltammetry (CV) cycling. As shown in Figure 3a, compared with the other catalysts, YS‐FMO_600_ exhibits the lowest overpotential of 248 mV at 10 mA cm^−2^. This performance is also among the highest values reported for Mo‐based OER catalysts (Table S4) and a similar trend was observed at a higher current density of 100 mA cm^−2^. It was deduced that YS‐FMO_600_ delivered an overpotential of 294 mV, while the CS‐FMO_500_, YS‐FMO_700_, and MoO_*x*_ catalysts exhibited overpotentials of 308, 310, and 400 mV, respectively, thereby highlighting the superior OER performance of the YS‐FMO_600_ system at high current densities (Figure 3b). Furthermore, YS‐FMO_600_ exhibited the lowest Tafel slope (51 mV dec^−1^) among the investigated catalysts, reflecting its favorable electrochemical kinetics during the OER (Figure 3c). Moreover, EIS was performed at 1.6 V_RHE_ to analyze the enhanced charge transfer at the electrolyte–catalyst interface of the electrocatalyst (Figure 3d).The measured charge transfer resistances (*R*_ct_) of the YS‐FMO_600_, CS‐FMO_500_, YS‐FMO_700_, and MoO_*x*_ systems were determined to be 0.41, 0.46, 0.43, and 1.67 Ω, respectively. This indicates that the FMO series catalysts exhibit significantly lower *R*_ct_ values than MoO_*x*_, which suggests that Fe substitution effectively promotes electrochemical charge transfer in MoO_*x*_ catalysts. To determine the intrinsic active sites, FeO_*x*_ was synthesized following the same procedure as that described for MoO_*x*_ and its electrocatalytic performance was evaluated. As shown in Figure S8a, FeO_*x*_ exhibited a low catalytic activity, with overpotentials of 341 mV at 10 mA cm^−2^ and 489 mV at 100 mA cm^−2^. Furthermore, EIS analysis revealed that FeO_*x*_ displays a relatively high charge–transfer resistance, suggesting that single‐metal active sites alone are insufficient to drive efficient OER kinetics (Figure S8b). These results confirm that the origin of the actual catalytic activity stems not from Fe or Mo sites, but from Fe–O–Mo hetero‐sites formed by bimetallic interactions between the two metals. The electrochemical surface area of the catalyst was characterized by the double‐layer capacitance (*C*_dl_). The FMO series exhibited increasing *C*_dl_ values with rising annealing temperatures, consistent with the structural evolution from a core–shell to a yolk–shell morphology (Figures S9–S10). In contrast, CS‐FMO_500_ exhibited a lower *C*_dl_ value than MoO_*x*_. This behavior was likely caused by its incompletely separated core–shell structure, in which the inner shell is not fully exposed as an active surface, together with the reduced porosity that limits electrolyte accessibility.

A key consideration toward the broad‐scale application of molybdenum‐based OER catalysts is overcoming their low stability under high‐current‐density conditions [70, 71]. Therefore, the durability of each prepared catalyst was evaluated at 100 mA cm^−2^ using CV and chronopotentiometry (CP). After 5000 cycles of accelerated durability testing (Figure 3e), the potential increased by 8 and 40 mV for YS‐FMO_600_ and MoO_*x*_, respectively. This result suggests that the electrochemical durability of YS‐FMO_600_ is superior to that of MoO_*x*_. Additionally, the long‐term performance was assessed at a high current density of 100 mA cm^−2^ to evaluate the catalyst stability (Figure 3f) [13, 72]. The potential of MoO_*x*_ sharply increased after ∼50 h, resulting in a total increase of 6.6%. In contrast, YS‐FMO_600_ maintained a negligible change of 0.6% over 100 h, demonstrating excellent electrochemical durability even under high current density conditions. After the stability test, we investigated the local atomic distribution and electronic structure of YS‐FMO_600_. Inductively coupled plasma optical emission spectroscopy (ICP‐OES) analysis revealed that MoO_*x*_ undergoes significant Mo dissolution under high‐current OER operating conditions. In contrast, YS‐FMO_600_ showed that Fe was largely retained during operation, while Mo partially dissolved (∼39%) under the same high‐current OER conditions (Table S5). Furthermore, STEM–EDS elemental mapping confirmed that the yolk–shell structure of YS‐FMO_600_ was well preserved even after prolonged operation, with Fe, Mo, and O elements uniformly distributed throughout the particle (Figure S11). XPS analysis revealed an increase in higher surface valence states of Mo and Fe due to oxidative reactions under OER conditions and partial Mo dissolution (Figure S12a–b) [30, 73]. In the O 1s spectra, a significant increase in the hydroxyl‐related (M–OH) component was observed, which can be interpreted as the occupation of vacancy sites generated during OER‐induced surface reconstruction by hydroxyl groups (Figure S12c) [74]. Importantly, this moderate Mo loss does not indicate degradation of the catalyst but rather reflects a controlled restructuring process in which a portion of Mo is removed while a significant fraction remains strongly coordinated with Fe and O. These results indicate that the Fe–Mo–O hetero‐interface is not completely destroyed during operation but instead evolves into a defect‐rich, Mo‐influenced interfacial structure. The persistent Fe–Mo–O interactions enable YS‐FMO_600_ to maintain a low degradation rate and high catalytic activity under high‐current operating conditions, while providing a rational explanation for the inferior performance of single‐component catalysts such as MoO_*x*_ and FeO_*x*_.

The overall water‐splitting performance of YS‐FMO_600_ was evaluated in a two‐electrode H‐type cell using a 1 M KOH electrolyte. As shown in Figure S13a, YS‐FMO_600_ required a cell voltage of 1.58 V to reach a current density of 10 mA cm^−2^. Furthermore, stability testing conducted at a current density of 100 mA cm^−2^ for 50 h showed that the cell voltage stabilizes at approximately 2.3 V during continuous operation (Figure S13b).

**FIGURE 4:**
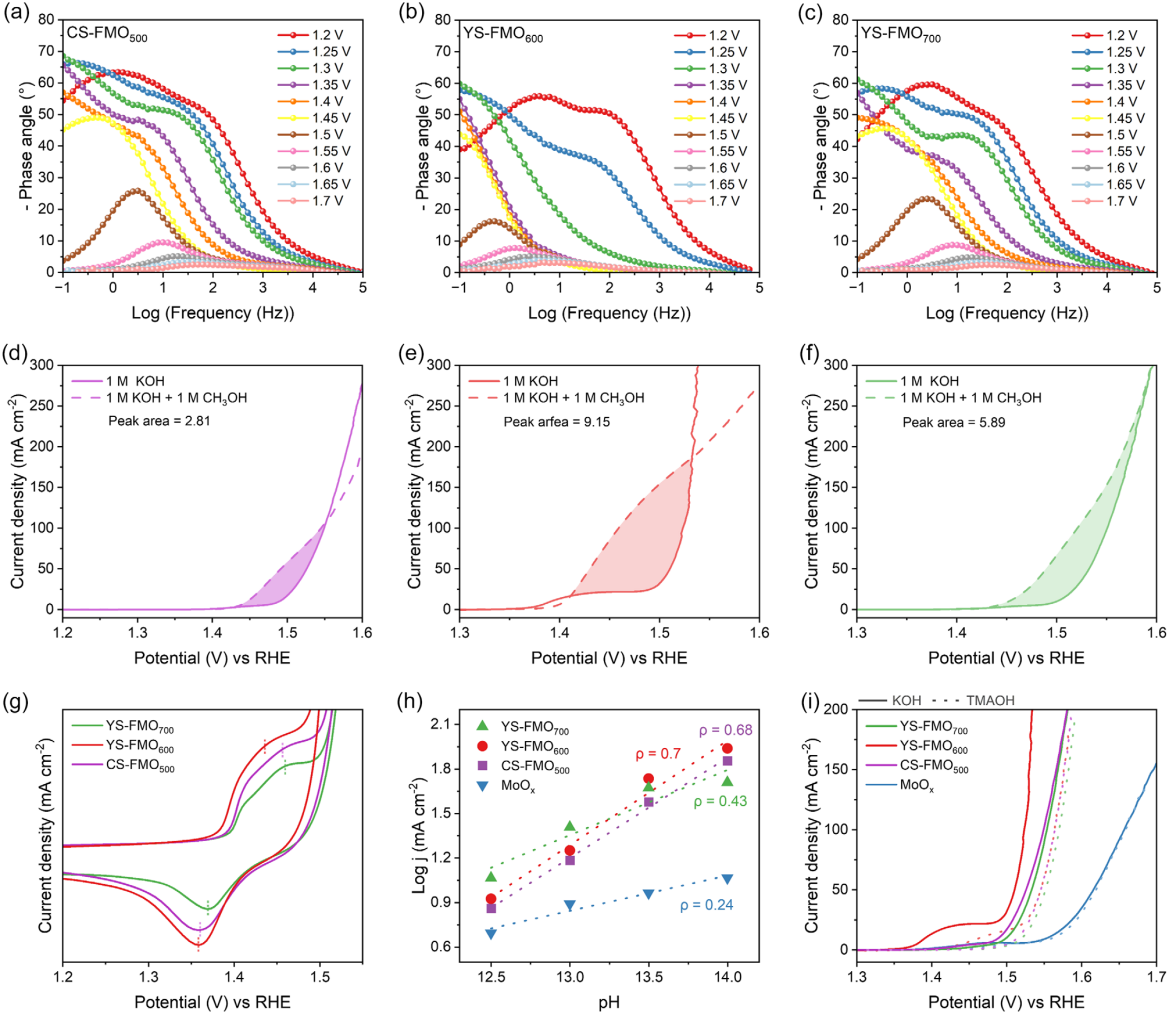
In situ EIS Bode plots recorded for (a) CS‐FMO_500_, (b) YS‐FMO_600_, and (c) YS‐FMO_700_. LSV curves for (d) CS‐FMO_500_, (e) YS‐FMO_600_, and (f) YS‐FMO_700_, recorded in 1 M KOH and 1 M KOH + 1 M methanol electrolytes. (g) CV curves and (h) pH dependence of the OER activity at 1.53 V. The proton reaction orders are derived from the slopes (*ρ*_RHE _=  ∂log j/∂pH). (i) LSV curves recorded in 1 M KOH and TMAOH electrolytes.

The enhanced OER activities and reaction mechanisms associated with the CS‐FMO_500_, YS‐FMO_600_, and YS‐FMO_700_ systems were investigated using in situ EIS to demonstrate the charge–transfer behaviors of the active species at the electrode–electrolyte interfaces. All catalysts showed a gradual decrease in the phase angle with increasing potential, indicating improved OER kinetics (Figure 4a–c). Notably, YS‐FMO_600_ exhibited a sharp decrease in its phase angle at a lower potential (1.3 *V*_RHE_) compared with those exhibited for the CS‐FMO_500_ and YS‐FMO_700_ catalysts, suggesting that charge transfer is significantly accelerated even at a relatively low overpotential [20, 62]. YS‐FMO_600_ displayed the lowest phase angle in the region prior to the onset potential (Figure S14). This was attributed to enhanced oxygen intermediate adsorption during the electrochemical reaction, facilitated by the abundant O_v_ generated during synthesis [75, 76].

The adsorption of oxygen intermediates was quantified through LSV measurements performed in a mixed electrolyte of 1 M methanol and 1 M KOH. By exploiting the nucleophilic nature of the methanol molecules, the *OH surface coverage was indirectly estimated based on the difference in the current response between the mixed electrolyte and 1 M KOH [57, 77, 78]. All catalysts demonstrated significant increases in their current densities in the presence of methanol, with the corresponding *OH coverage increasing in the order of CS‐FMO_500_ < YS‐FMO_700_ < YS‐FMO_600_ (Figure 4d–f). This result emphasizes the excellent OER reaction kinetics of YS‐FMO_600_ and accounts for the accelerated charge transfer observed by in situ EIS.

To further clarify the adsorption characteristics of the oxygen intermediates, CV was performed in a 1 M KOH electrolyte to examine the redox current. As previously reported, oxygen insertion coupled with Mo oxidation gives rise to an oxidation peak at 1.43–1.47 *V*_RHE_ under OER‐relevant conditions [22]. Among the current systems, YS‐FMO_600_ exhibited larger redox currents than its counterparts (Figure 4g), thereby implying that the abundant O_v_ generated during annealing provides favorable sites for active oxygen incorporation. In addition, the negative shift of the redox peaks in YS‐FMO_600_ indicates faster adsorption of oxygen intermediates at lower oxidation potentials, reflecting improved adsorption/desorption kinetics [79, 80].

Moreover, the introduction of Fe demonstrates the potential to activate the LOM pathway by inducing the electronic restructuring of Mo and enhancing metal–oxygen orbital hybridization [7, 22, 30]. To investigate this effect, the OER activities of YS‐FMO_600_, CS‐FMO_500_, and YS‐FMO_700_ were measured under various pH conditions (pH 12.5–14). Notably, a pH‐dependent activity originates from the kinetic differences between electron and proton transfer, which serves as a useful approach to differentiate OER mechanisms [81]. As shown in Figures 4h and S15, the pH‐dependent reaction sensitivity increases in the order MoO_*x*_ < CS‐FMO_500_ < YS‐FMO_700_ < YS‐FMO_600_, and the proton reaction order of YS‐FMO_600_ was determined to be 0.7, indicating the strongest pH dependence [82]. This result aligns with the characteristic nonconcerted proton–electron transfer process typically observed in LOM pathways [83, 84].

To further elucidate the involvement of the LOM pathway, a chemical probe experiment was performed using an electrolyte containing the tetramethylammonium cation (TMA^+^). Oxygen intermediates such as O_2_^2−^ and O_2_ (peroxo/superoxo‐like species) generated in the LOM pathway interact strongly with TMA^+^. This significantly interferes with the OER kinetics and can be used as an indicator to indirectly evaluate the contribution of the LOM pathway [77, 85]. Figure 4i shows that YS‐FMO_600_ undergoes a pronounced decrease in current density in the 1 M TMAOH (tetramethylammonium hydroxide) electrolyte, corresponding to an activity loss of ∼ 81.6% at 1.53 V_RHE_. This behavior indicates that oxygen evolution in YS‐FMO_600_ is effectively inhibited by TMA^+^, further confirming the active involvement of the LOM pathway. These insights imply that the introduction of Fe plays a key role in shifting the OER pathway in MoO_*x*_ from the conventional adsorbate evolution mechanism to the LOM. Moreover, the obtained results further suggest that this transition is strongly modulated by the degree of lattice distortion and the O_v_ concentration.

## 3 Experimental

### 3.1 Synthesis of the MoO*_x_* and FMO Specimens (YS‐FMO_600_, CS‐FMO_500_, and YS‐FMO_700_)

The MoO_*x*_ and FMO series specimens were synthesized via spray pyrolysis. Spray precursor solutions were prepared by dissolving 0.05 M ammonium molybdate tetrahydrate ((NH_4_)_6_Mo_7_O_24_·4H_2_O, 99%, Sigma–Aldrich) for MoO_*x*_ or a mixture of 0.025 M ammonium molybdate tetrahydrate ((NH_4_)_6_Mo_7_O_24_·4H_2_O, 99%, Sigma–Aldrich) and 0.025 M iron(II) acetate tetrahydrate (Fe(CO_2_CH_3_)_2_·4H_2_O, 95%, Sigma–Aldrich) for the FMO series, in distilled water. Subsequently, 0.1 M citric acid (HOC(COOH)(CH_2_COOH)_2_, 99%, Sigma–Aldrich) was added as a complexing agent and pH adjuster, and each mixture was stirred at 400 rpm for 1 h to produce uniform dispersions. Each mixed precursor solution was subsequently atomized into droplets using an ultrasonic nebulizer (frequency: 1.7 MHz). The droplets were then introduced into a quartz tube reactor under a flow of nitrogen gas (10 L min^−1^) to perform the pyrolysis reaction. For this purpose, a tube furnace (Lindberg/Blue M, Thermo Scientific) was set to 600°C for the synthesis of MoO_*x*_ and YS‐FMO_600_, while temperatures of 500°C and 700°C were employed to prepare CS‐FMO_500_ and YS‐FMO_700_, respectively. The resulting products were collected as powders on a back filter (Teflon, pore size: 0.2 μm).

### 3.2 Material Characterization

The morphologies and structures of the catalysts were observed using FE‐SEM (MIRA3 LM, TESCAN) and FE‐TEM (JEM‐F200, JEOL) at an accelerating voltage of 200kV. HAADF–STEM and HRTEM were also performed using the same JEM‐F200 instrument. XRD (Ultima IV, Rigaku) was performed in the *θ*–2*θ* mode over a 2*θ* range of 10°–70° using Cu K*α* radiation (*λ* = 1.541 Å). Raman spectroscopy (inVia Raman Microscope, Renishaw, *λ* = 532 nm) was conducted to identify the composite oxide phases and examine the effects of Fe substitution. To observe the surface oxidation states, XPS (Axis‐Supra, Kratos) was performed using an Al K*α* source. All XPS spectra were calibrated at 284.6 eV using the C 1s line. The binding energy difference between the Mo 3d_5/2_ and Mo 3d_3/2_ peaks was determined to be 3.1 eV by peak fitting, while that between the Fe 2p_3/2_ and Fe 2p_1/2_ peaks was 13.1 eV. Additionally, EPR spectroscopy (JES‐X320, JEOL) was performed to detect the O_v_. BET analysis was carried out using a surface area and porosimetry system (BELSORP‐max, MicrotracBEL) to determine the specific surface areas and pore structures of the catalysts. UV–vis spectrophotometry (LAMBDA 1050, PerkinElmer, 200–800 nm) was performed to construct Tauc plots for estimation of the bandgap energies.

### 3.3 Electrochemical Measurements

The electrochemical performances of the synthesized catalysts were evaluated using an electrochemical workstation (WaveDriver 100, PINE research) with a 1 M KOH electrolyte. All electrochemical measurements were conducted in a three‐electrode system, using Ni foam loaded with the synthesized catalyst as the working electrode, Pt mesh as the counter electrode, and Hg/HgO (1.0 M NaOH) as the reference electrode. In all experiments, the total catalyst loading was fixed at 25 mg cm^−2^ to ensure sufficient catalyst coating on the Ni foam support. The catalyst was then dispersed in a mixture of ethanol (425 μL) and a Nafion solution (20%, 75 μL) to form a uniform catalyst ink. Subsequently, each ink was drop‐cast onto the surface of the Ni foam and dried under air to preparethe working electrodes. All potentials were converted to reversible hydrogen electrode (RHE) equivalents using the following equation:

$$E_{\mathrm{RHE}}=E_{\mathrm{Hg}/\mathrm{HgO}}+0.0592\;\times \;\mathrm{pH}+0.098$$

To minimize contributions from redox processes and capacitive currents, the catalytic activity was evaluated using backward LSV recorded at a scan rate of 1 mV s^–1^. All potentials were corrected for iR compensation. EIS was conducted between 0.01 Hz and 1 MHz at 1.6 *V*_RHE_. *C*_dl_ analysis was performed using CV scans recorded at a scan rate of 20–100 mV s^–1^ over a potential range of 0.87−1.03 *V*_RHE_. Accelerated durability tests were performed for the OER over 5000 CV scans within the potential range of 1.15–1.8 *V*_RHE_ at a scan rate of 200 mV s^−1^. The long‐term stability of each catalyst was evaluated by CP at 100 mA cm^–2^ for 100 h. In situ EIS was performed over a frequency range of 0.1–10^5^ Hz with an amplitude of 10 mV at various applied potentials for the OER. The pH‐dependence and TMAOH‐probe LSV measurements were performed in pH‐adjusted KOH electrolytes and in 1 M TMAOH (25 wt% solution in H_2_O, Sigma–Aldrich), respectively, at a scan rate of 10 mV s^−1^. In the two‐electrode H‐cell configuration, YS‐FMO_600_ electrodes and a Pt foil were used as the anode and cathode, respectively, with a Fumasep FAA‐3‐50 membrane employed as the separator.

## 4 Conclusion

In this study, a series of Fe‐substituted MoO_*x*_ catalysts was prepared via a one‐step spray pyrolysis method, and the impacts of both Fe substitution and the annealing temperature on their structural, electronic, and electrocatalytic properties were systematically investigated. Fe substitution not only increased the specific surface area of MoO_*x*_, but also modulated its electronic structure, resulting in a narrowed bandgap and significantly enhanced OER activity and electrochemical stability. In addition, control of the annealing temperature effectively regulated lattice distortion and O_v_ concentration. The optimized YS‐FMO_600_ catalyst exhibited a reaction mechanism consistent with the LOM, which was driven by the enhanced adsorption of oxygen intermediates, and promoted O—O bond formation involving lattice oxygen species. These findings identify electronic structure modulation and lattice oxygen activation as general design principles for developing efficient and durable OER catalysts, which may be extended to the engineering of other Mo‐based and transition metal oxides. Overall, this study provides valuable guidance for the rational design of cost‐effective and high‐performance electrocatalysts.

## Supporting Information

Additional supporting information can be found online in the Supporting Information Section. **Supporting Fig. S1:** (a) Photographic images and (b) TEM images of MoO_x_, CS‐FMO_500_, YS‐FMO_600_, and YS‐FMO_700_. **Supporting Fig. S2:** (a) TEM image and (b) corresponding STEM‐EDS elemental mapping images of CS‐FMO_500_, showing the distributions of Mo, Fe, and O. **Supporting Fig. S3:** N_2_ adsorption/desorption isotherms and corresponding pore size distributions (insets) of (a) MoO_x_ and (b) YS‐FMO_600_. (c) Specific surface areas and pore sizes of MoO_x_ and YS‐FMO_600_. **Supporting Fig. S4:** XRD pattern of MoO_x_ along with the reference diffraction patterns of MoO_3_ and MoO_2_. **Supporting Fig. S5:** HRTEM images recorded for (a) MoO_x_, (b) CS‐FMO_500_, (c) YS‐FMO_600_, and (d) YS‐FMO_700_. **Supporting Fig. S6:** Effect of the annealing temperature on the Mo^4+^/Fe^3+^ ratio and O_v_ content of YSFMO_600_. **Supporting Fig. S7:** (a) UV‐vis absorbance spectra and (b) Tauc plots for estimation of the band gap energies of MoO_x_ and YS‐FMO_600_. **Supporting Fig. S8:** (a) LSV curve with iR correction, and (b) EIS spectrum recorded at 1.6 V_RHE_ for FeO_x_. **Supporting Fig. S9:** C_dl_ measurements performed for MoO_x_, CS‐FMO_500_, YS‐FMO_600_, and YSFMO_700_ (units: mF cm^−2^). **Supporting Fig. S10:** CV curves recorded for the C_dl_ calculations performed in 1 M KOH using (a) MoO_x_, (b) CS‐FMO_500_, (c) YS‐FMO_600_, and YS‐FMO_700_. **Supporting Fig. S11:** TEM image and corresponding STEM‐EDS elemental mapping images of YSFMO_600_ after the stability test. **Supporting Fig. S12**: XPS spectra recorded for the (a) Mo 3d, (b) Fe 2p, and (c) O 1s components of YS‐FMO_600_ after the stability test. **Supporting Fig. S13:** Electrocatalytic OER performance of YS‐FMO_600_ in a 1 M KOH electrolyte measured in an H‐type cell. (a) LSV curve and (b) CP results obtained at 100 mA cm^−2^ for 50 h. **Supporting Fig. S14:** Phase angle variation at 0 Hz and various applied potentials for CS‐FMO_500_, YS‐FMO_600_, and YS‐FMO_700_. **Supporting Fig. S15:** Effect of pH on the catalytic performances of (a) MoO_x_, (b) CS‐FMO_500_, (c) YS‐FMO_600_, and (d) YS‐FMO_700_. **Supporting Table S1:** Deconvoluted XPS data for Mo 3d showing the binding energies and relative intensities for the various oxidation states. **Supporting Table S2:** Deconvoluted XPS data for Fe 2p showing the binding energies and relative intensities for the various oxidation states. **Supporting Table S3:** Deconvoluted XPS data for O 1s showing the binding energies and relative intensities for the various oxidation states. **Supporting Table S4:** Comparison of the OER performance between YS‐FMO_600_ and previously reported Mo‐based electrocatalysts. **Supporting Table S5:** ICP‐OES comparison of MoO_x_ and YS‐FMO_600_ before and after the stability test.

## Conflicts of Interest

The authors declare no conflicts of interest.

## Supporting information

Supplementary Material

## Data Availability

The data that support the findings of this study are available in the supplementary material of this article.
