# Supplementary material for: Fe‐Substituted MoO x Catalysts With Lattice Distortion–Vacancy Coupling for Enhanced Alkaline Oxygen Evolution Reaction
Source: ChemSusChem. 2026 Feb 12;19(3):e202502390. doi: 10.1002/cssc.202502390 (PMC12900164; doi:10.1002/cssc.202502390)
Supplement: Supplementary file 1 — Supplementary Material [file CSSC-19-e202502390-s001.pdf]

Supporting Information

Fe-Substituted MoO_x Catalysts with Lattice Distortion–Vacancy Coupling for Enhanced Alkaline Oxygen Evolution Reaction

*Minhui Kim,[a] Byounguk Yu,[a] Hye Young Koo,[b] Yuchan Kim,[b] and Dahee Park[a]**

[a] M. Kim, B. Yu, D. Park
Energy and Environment Materials Research Division, Korea Institute of Materials Science (KIMS), Changwon 51508, Republic of Korea

E-mail: daheepark@kims.re.kr (D. Park)

[b] H. Y. Koo, Y. Kim
Nano Materials Research Division, Korea Institute of Materials Science (KIMS), Changwon 51508, Republic of Korea

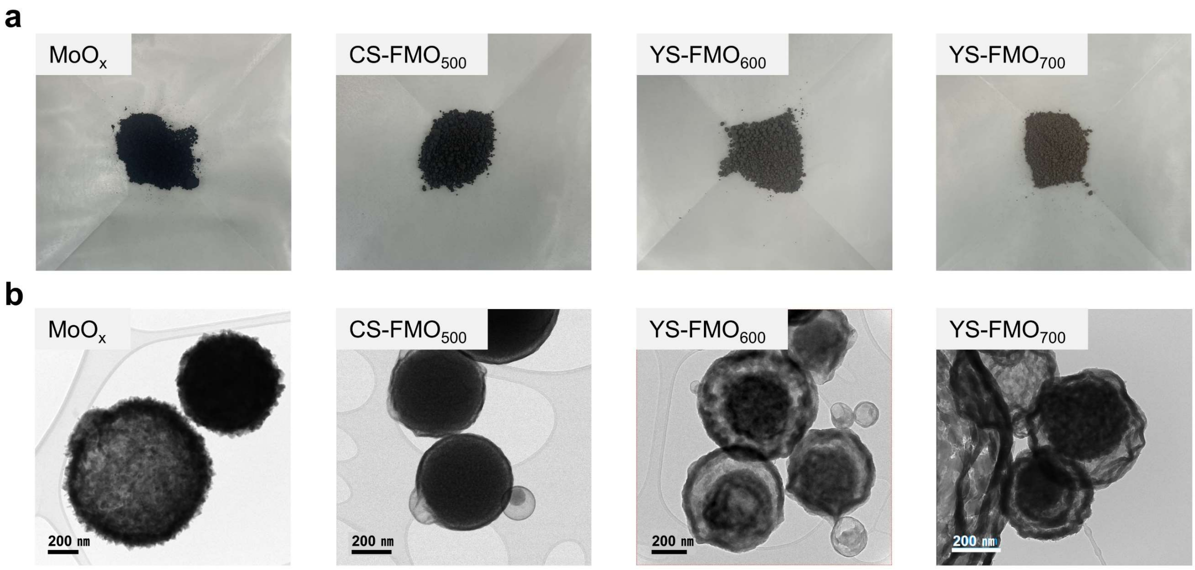

Figure S1. (a) Photographic images and (b) TEM images of MoO_x, CS-FMO_{500}, YS-FMO_{600}, and YS-FMO_{700}.

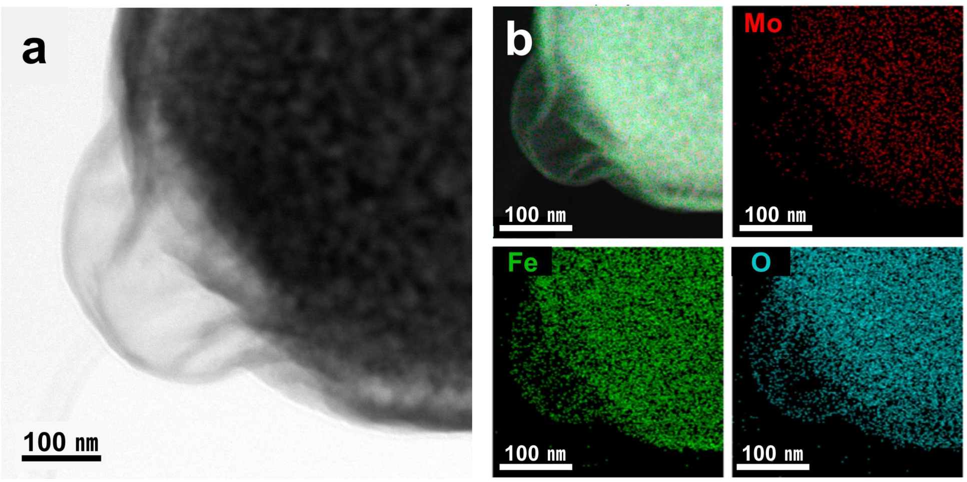

Figure S2. (a) TEM image and (b) corresponding STEM-EDS elemental mapping images of CS-FMO_{500}, showing the distributions of Mo, Fe, and O.

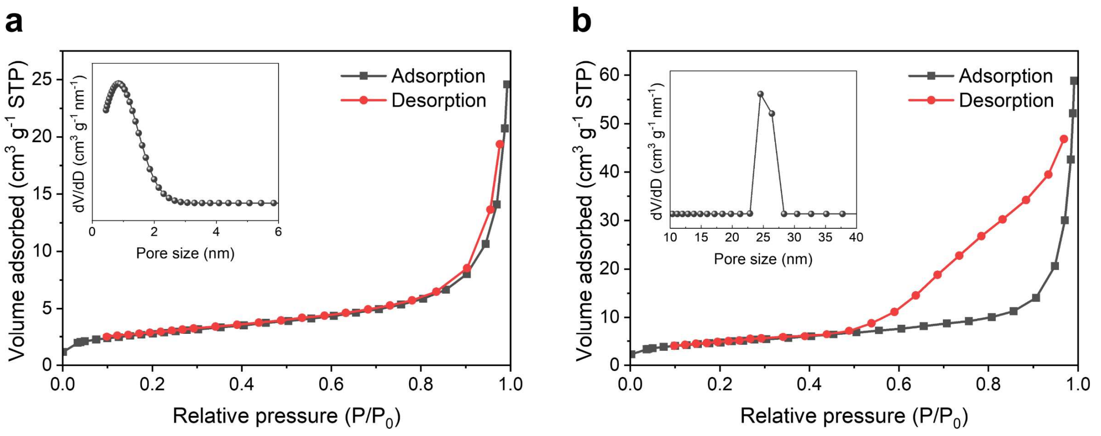

c

Sample	MoO_x	YS-FMO_{600}
BET surface area (m^2 g^{-1})	9.89	17.00
Pore size (nm)	0.73	24.56

Figure S3. N_2 adsorption/desorption isotherms and corresponding pore size distributions (insets) of (a) MoO_x and (b) YS-FMO_{600}. (c) Specific surface areas and pore sizes of MoO_x and YS-FMO_{600}.

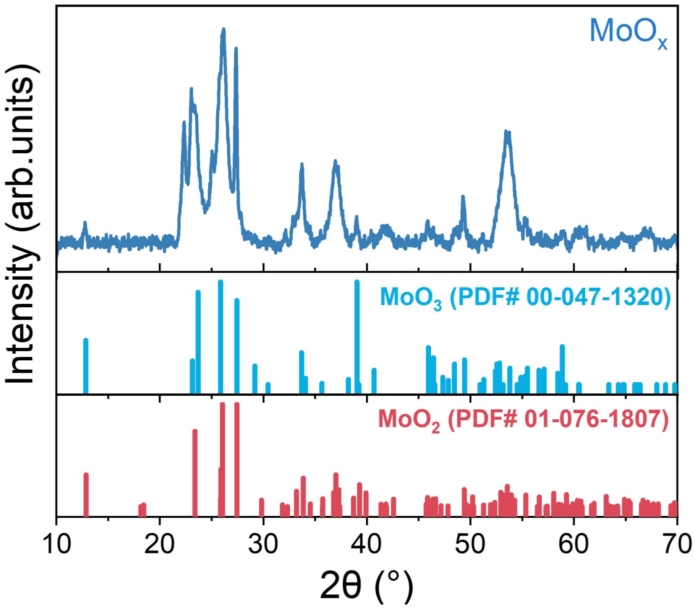

Figure S4. XRD pattern of MoO_x along with the reference diffraction patterns of MoO_3 and MoO_2.

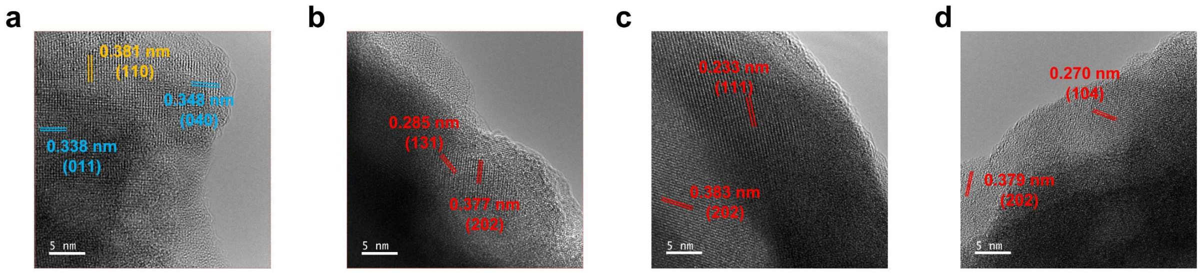

Figure S5. HRTEM images recorded for (a) MoO_x, (b) CS-FMO_{500}, (c) YS-FMO_{600}, and (d) YS-FMO_{700}.

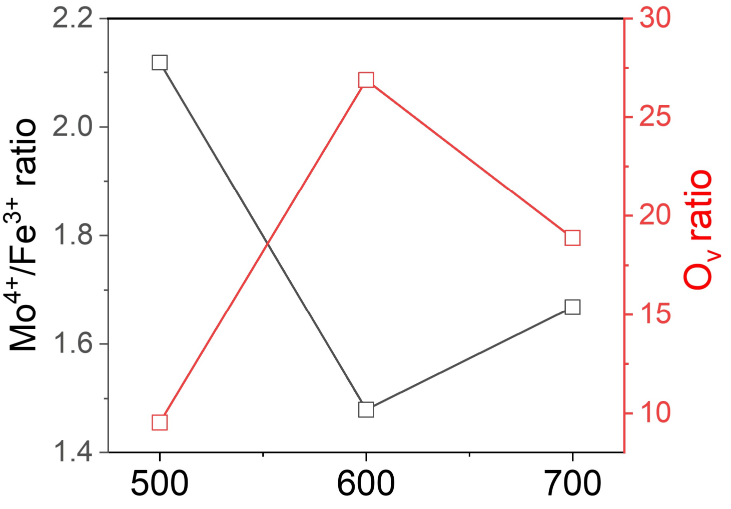

Figure S6. Effect of the annealing temperature on the Mo^{4+}/Fe^{3+} ratio and O_v content of YS-FMO$_{600}$.

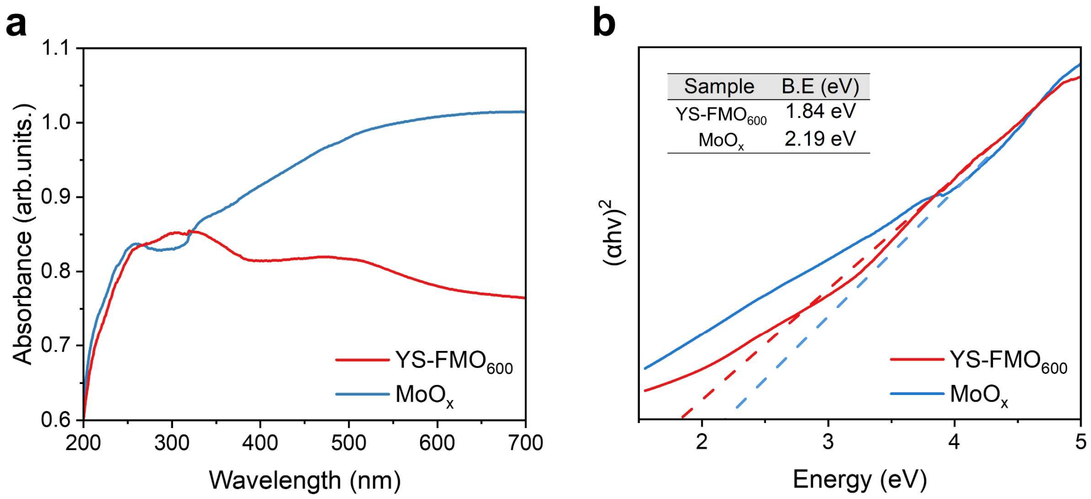

Figure S7. (a) UV-vis absorbance spectra and (b) Tauc plots for estimation of the band gap energies of MoO_x and YS-FMO_{600}.

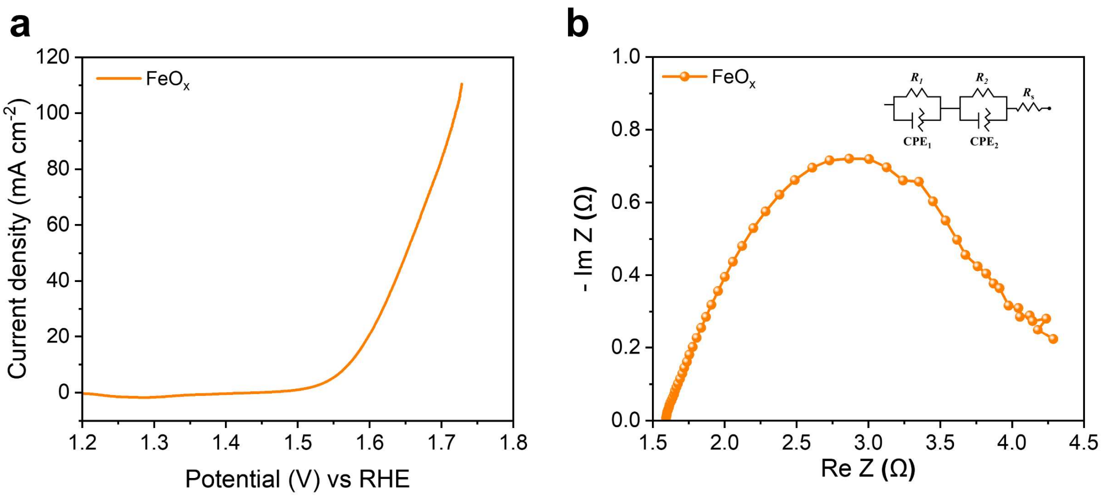

Figure S8. (a) LSV curve with iR correction, and (b) EIS spectrum recorded at 1.6 V_{RHE} for FeO_x.

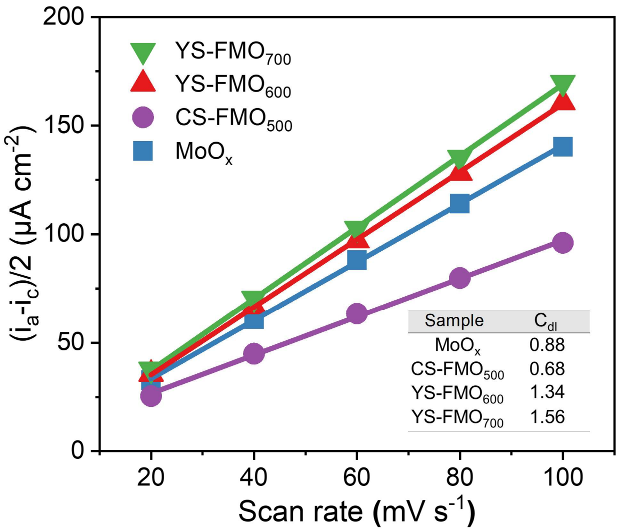

Figure S9. C_{dl} measurements performed for MoO_x, CS-FMO_{500}, YS-FMO_{600}, and YS-FMO_{700} (units: mF cm^{-2}).

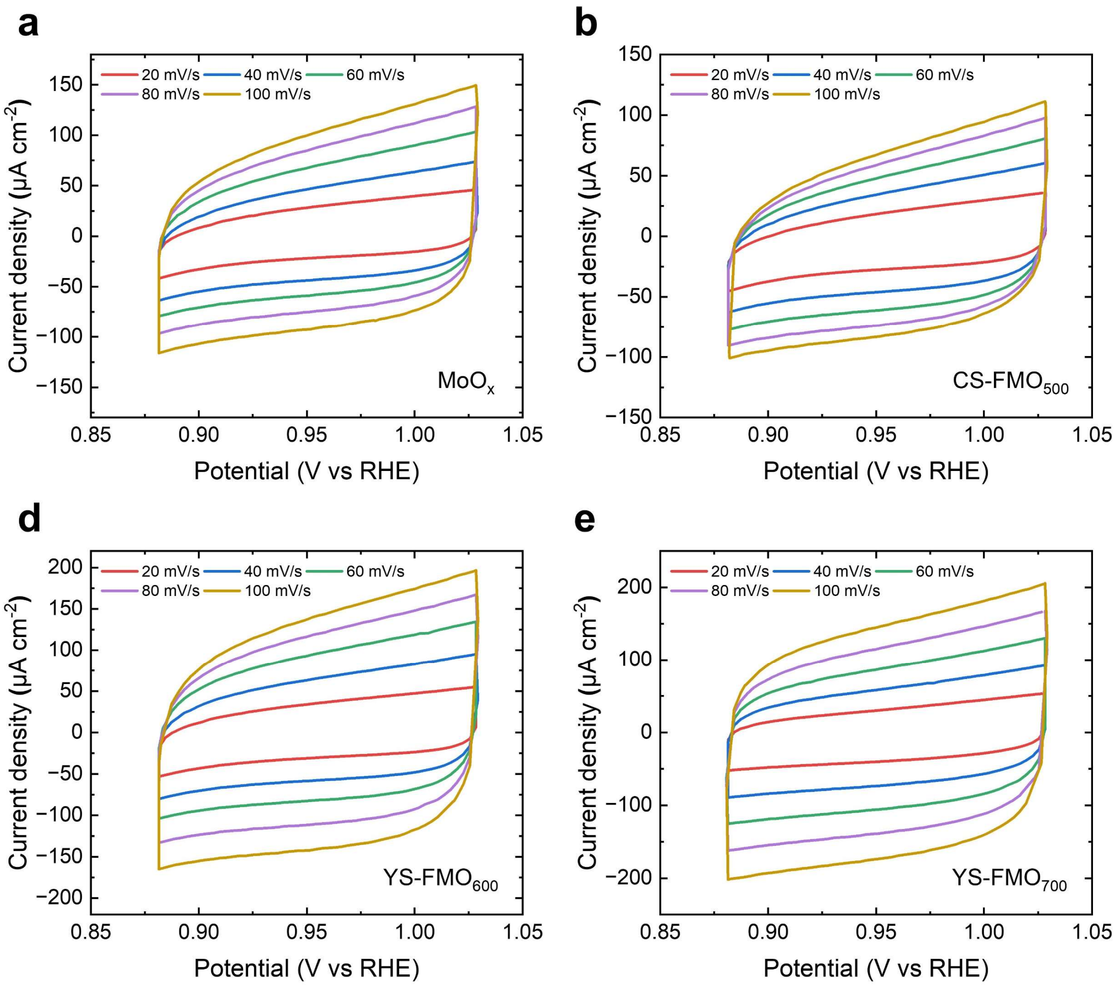

Figure S10. CV curves recorded for the C_{dl} calculations performed in 1 M KOH using (a) MoO_x, (b) CS-FMO_{500}, (c) YS-FMO_{600}, and YS-FMO_{700}.

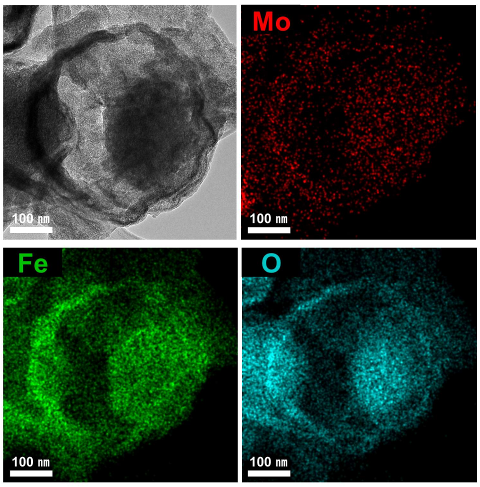

Figure S11. TEM image and corresponding STEM-EDS elemental mapping images of YS-FMO_{600} after the stability test.

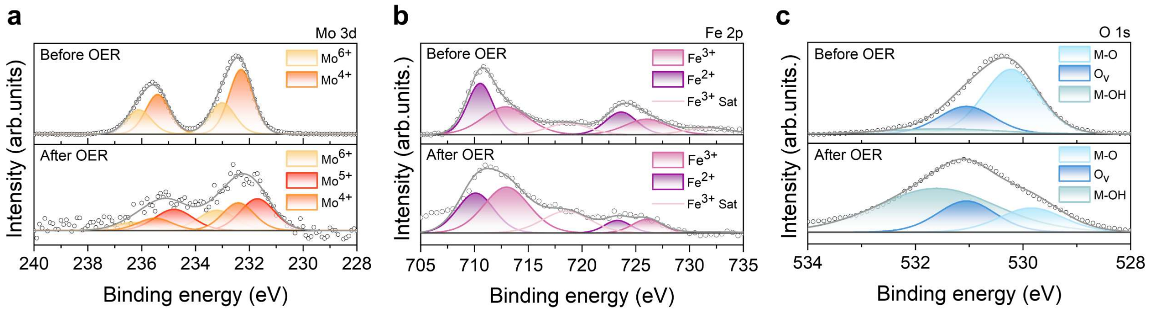

Figure S12. XPS spectra recorded for the (a) Mo 3d, (b) Fe 2p, and (c) O 1s components of YS-FMO_{600} after the stability test.

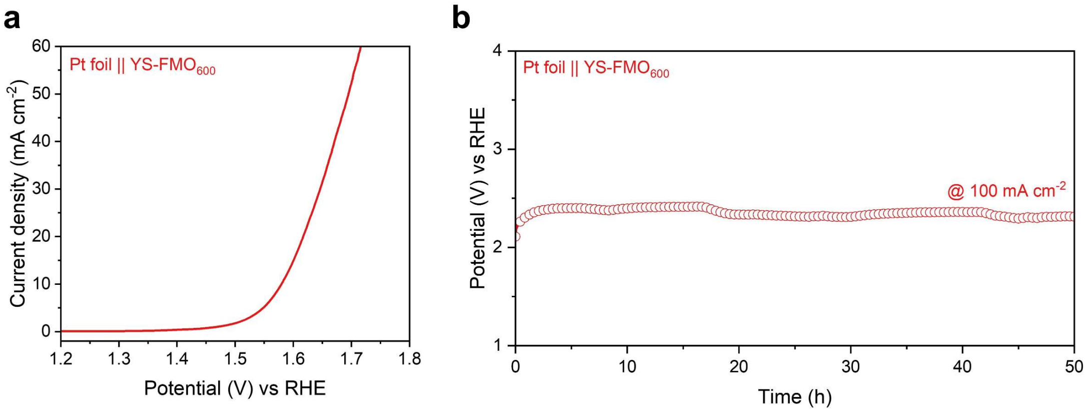

Figure S13. Electrocatalytic OER performance of YS-FMO_{600} in a 1 M KOH electrolyte measured in an H-type cell. (a) LSV curve and (b) CP results obtained at 100 mA cm^{-2} for 50 h.

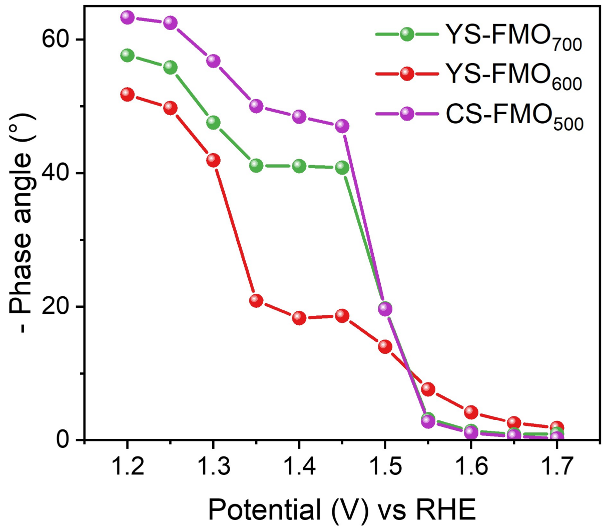

Figure S14. Phase angle variation at 0 Hz and various applied potentials for CS-FMO_{500}, YS-FMO_{600}, and YS-FMO_{700}.

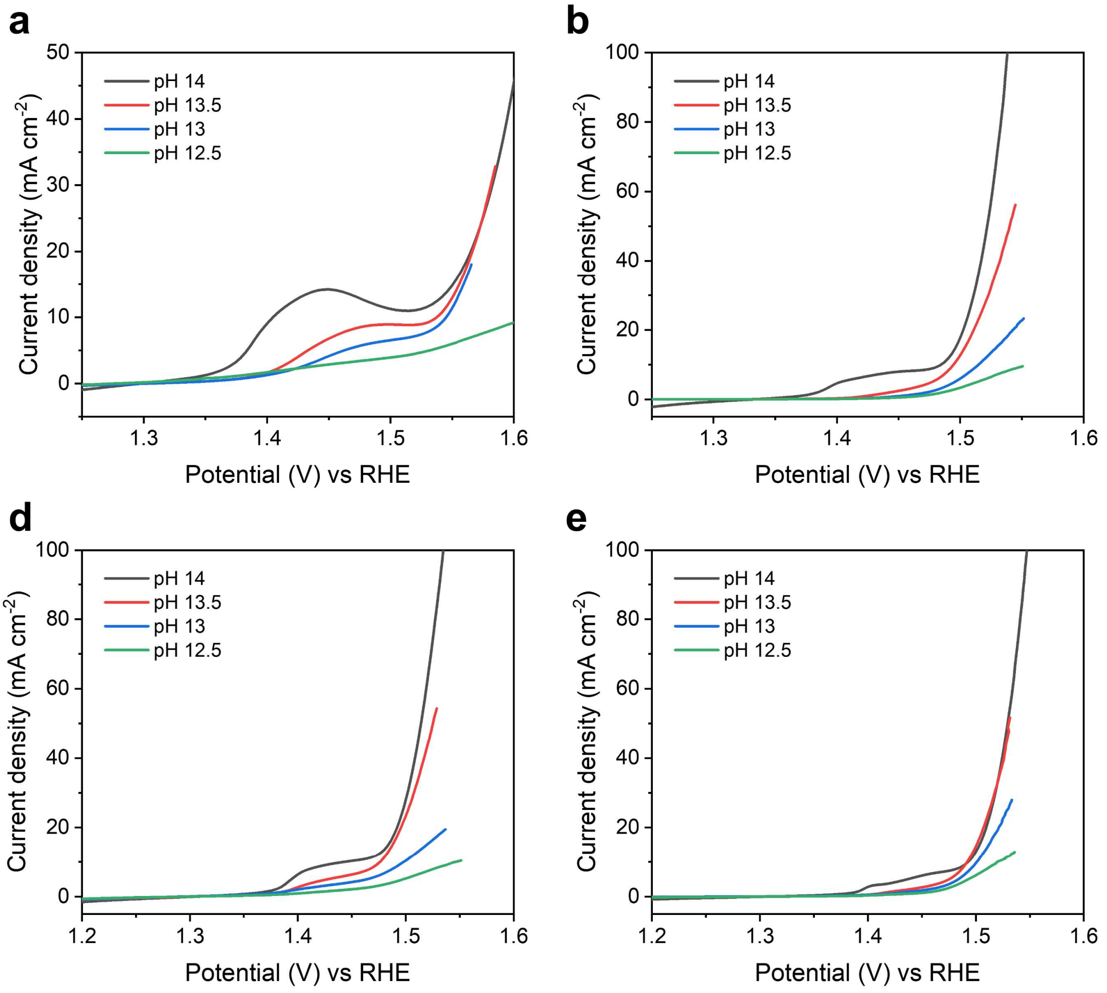

Figure S15. Effect of pH on the catalytic performances of (a) MoO_x, (b) CS-FMO$_{500}$, (c) YS-FMO$_{600}$, and (d) YS-FMO$_{700}$.

Table S1. Deconvoluted XPS data for Mo 3d showing the binding energies and relative intensities for the various oxidation states

Catalyst	Peak	Oxidation state	Binding energy (eV)	Ratio (%)
YS-FMO_{700}	Mo 3d	6	233.0	23.7
		4	232.4	37.7
YS-FMO_{600}		6	233.0	20.3
		4	232.3	39.8
CS-FMO_{500}		6	233.0	1.7
		4	232.3	59.1
MoO_x		6	233.1	33.8
		4	232.3	23.4

Table S2. Deconvoluted XPS data for Fe 2p showing the binding energies and relative intensities for the various oxidation states

Catalyst	Peak	Oxidation state	Binding energy (eV)	Ratio (%)
$YS\text{-}FMO_{700}$	Fe 2p	3	712.8	22.6
		2	710.6	30.2
$YS\text{-}FMO_{600}$	Fe 2p	3	712.9	26.9
		2	710.5	27.9
$CS\text{-}FMO_{500}$	Fe 2p	3	712.7	27.9
		2	710.6	23.0

Table S3. Deconvoluted XPS data for O 1s showing the binding energies and relative intensities for the various oxidation states

Catalyst	Peak	Oxidation state	Binding energy (eV)	Ratio (%)
YS-FMO700	O 1s	M-O	530.2	70.8
		O_v	531.0	18.9
		M-OH	531.5	10.4
YS-FMO600		M-O	530.2	63.0
		O_v	531.1	26.9
		M-OH	531.5	10.2
CS-FMO500		M-O	530.1	82.0
		O_v	531.1	9.5
		M-OH	531.5	8.5
MoO_x		M-O	530.2	3.7
		O_v	531.0	43.9
		M-OH	531.5	52.5

Table S4. Comparison of the OER performance between YS-FMO_{600} and previously reported Mo-based electrocatalysts

Catalysts	Electrolyte	Substrate	η_{10} (mV)	Tafel slope (mV dec^{-1})	Stability	Ref.
**YS-FMO_{600}**	**1 M KOH**	**Ni foam**	**248**	**51**	**100 h @ 100 mA cm^{-2}**	**This work**
**MoO_x**	**1 M KOH**	**Ni foam**	**359**	**101**	**100 h @ 100 mA cm^{-2}**	**This work**
30-$CoMoO_4$	1 M KOH	Ni foam	253	58	5000 cycles	[20]
Ni-$FeNi_3$/$Ni_{0.5-b}Fe_{0.5-y}Mo_{1.5}O_x$	1 M KOH	Glassy carbon	278	42	30 h @ 10 mA cm^{-2}	[22]
RuMo-Air	1 M KOH	Carbon paper	260	52.8	10,000 cycles	[86]
Mo-CoOOH	1 M KOH	Glassy carbon	269	51.7	20 h @ 10 mA cm^{-2}	[87]
Vo-$CoMoO_4 \cdot nH_2O$@$Co(OH)_2$	1 M KOH	Glassy carbon	270	62.6	110 h @ 10 mA cm^{-2}	[88]
Mo_1-CoOOH@CP	1 M KOH	Carbon paper	274	66	100 h @ 10 mA cm^{-2}	[89]
P-Mo-Co_3O_4@CC	1 M KOH	Carbon cloth	276	53.9	27 h @ 10 mA cm^{-2}	[90]
0.5Mo-$NiCo_2O_4$	1 M KOH	Ni foam	280	43	24 h @ 10 mA cm^{-2}	[91]
CoMo hydr(oxy)oxide NS	0.1 M KOH	Glassy carbon	377	41.88	6 h @ 10 mA cm^{-2}	[92]
NMCP@NF	1 M KOH	Ni foam	250	41	30 h @ 50 mA cm^{-2}	[93]

Table S5. ICP-OES comparison of MoO_x and YS-FMO_{600} before and after the stability test

Sample	MoO_x		YS-FMO_{600}	
Status	Before reaction	After durability	Before reaction	After durability
Mo (ppm)	139,000	748	93,800	56,830
Fe (ppm)	-	-	56,700	56,100
Mo/Fe	-	-	1.65	1.01

Supporting Information References

[20] H. Xiao, K. Chi, H. Yin, X. Zhou, P. Lei, P. Liu, J. Fang, X. Li, S. Yuan, Z. Zhang, "Excess Activity Tuned by Distorted Tetrahedron in $CoMoO_4$ for Oxygen Evolution," *Energy Environ. Mater.* **2024,** *7* (1), e12495. https://doi.org/10.1002/eem2.12495.

[22] J. Fan, X. Zhang, M. Han, X. Xiang, C. Guo, Y. Lin, N. Shi, D. Xu, Y. Lai, J. Bao, "Amorphous Ni–Fe–Mo Oxides Coupled with Crystalline Metallic Domains for Enhanced Electrocatalytic Oxygen Evolution by Promoted Lattice-Oxygen Participation," *Small* **2024,** *20* (10), 2303927. https://doi.org/10.1002/smll.202303927.

[86] C. Li, J. Chen, K. C. Chong, L. Wang, B. Liu, "Tailoring the Chemical Environment of Ru–Mo Composites for Efficient Hydrogen and Oxygen Evolution Reactions," *Small Structures* **2024,** *5* (3), 2300394. https://doi.org/10.1002/sstr.202300394.

[87] Z. Jia, Y. Yuan, Y. Zhang, X. Lyu, C. Liu, X. Yang, Z. Bai, H. Wang, L. Yang, "Optimizing 3d Spin Polarization of CoOOH by In Situ Mo Doping for Efficient Oxygen Evolution Reaction," *Carbon Energy* **2024,** *6* (1), e418. https://doi.org/10.1002/cey2.418.

[88] K. Wang, Y. Li, J. Hu, Z. Lu, J. Xie, A. Hao, Y. Cao, "Deep Reconstruction of Transition Metal Molybdate@Hydroxide Heterostructure Triggered by Anion-Exchange Reaction as High Efficiency Water Oxidation Electrocatalyst," *Chem. Eng. J.* **2022,** *447*, 137540. https://doi.org/10.1016/j.cej.2022.137540

[89] Y. Zhang, H. Guo, P. Yuan, K. Pang, B. Cao, X. Wu, L. Zheng, R. Song, "Structural Evolution of $CoMoO_4$ to CoOOH by Ion Electrochemical Etching for Boosting Oxygen Evolution Reaction," *J. Power Sources* **2019,** *442*, 227252. https://doi.org/10.1016/j.jpowsour.2019.227252

[90] Y. Huang, M. Li, F. Pan, Z. Zhu, H. Sun, Y. Tang, G. Fu, "Plasma-Induced Mo-Doped Co_3O_4 with Enriched Oxygen Vacancies for Electrocatalytic Oxygen Evolution in Water Splitting," *Carbon Energy* **2023,** *5* (3), e279. https://doi.org/10.1002/cey2.279

[91] S. Xiong, L. Wang, H. Chai, Y. Xu, Y. Jiao, J. Chen, "Molybdenum Doped Induced Amorphous Phase in Cobalt Acid Nickel for Supercapacitor and Oxygen Evolution Reaction," *J. Colloid Interface Sci.* **2022,** *606*, 1695–1706. https://doi.org/10.1016/j.jcis.2021.08.151.

[92] S. Bera, W.-J. Lee, E.-K. Koh, C.-M. Kim, S. Ghosh, Y. Yang, S.-H. Kwon, "Enhancing Water Oxidation Activity by Tuning Two-Dimensional Architectures and

Compositions on CoMo Hydr(oxy)oxide," *J. Phys. Chem. C* **2020,** *124* (31), 16879–16887. https://doi.org/10.1021/acs.jpcc.0c01411

[93] M. R. Kandel, U. N. Pan, D. R. Paudel, P. P. Dhakal, N. H. Kim, J. H. Lee, "Hybridized Bimetallic Phosphides of Ni-Mo, Co-Mo, and Co-Ni in a Single Ultrathin-3D-Nanosheets for Efficient HER and OER in Alkaline Media," *Compos. B Eng.* **2022,** *239*, 109992. https://doi.org/10.1016/j.compositesb.2022.109992.
